# Supplementary material for: Heme-stress activated NRF2 skews fate trajectories of bone marrow cells from dendritic cells towards red pulp-like macrophages in hemolytic anemia
Source: Cell Death Differ. 2022 Jan 14;29(8):1450–65. doi: 10.1038/s41418-022-00932-1 (PMC9345992; doi:10.1038/s41418-022-00932-1)
Supplement: Supplementary file 1 — Supplementary figure legends [file 41418_2022_932_MOESM1_ESM.docx]

**SUPPLEMENTARY FIGURE LEGENDS**

**Supplementary Figure 1**

## Flow cytometry contour plots of GM-CSF- and M-CSF BM cultures. The cells were stained for CD11c, MHC class II, CD11b, and CD115.

## SnMP fluorescence intensities were measured in GM-CSF BM cultures after 3h incubation with the SnMP porphyrin (100 µM)using a SP6800 Spectral Analyzer with the 640 nm laser inactivated.. The spectral plots show fluorescence emission intensities in macrophages defined as CD115^+^ , MHC class II^low^ (boxed in blue) and in DCs defined as CD115^_^ MHC class II^high^(boxed in magenta). The emission spectrum in macrophages matches the emission spectrum of SnMP measured in solution. Excitation was at 405 nm for flow cytometry and for the in solution fluorescence emission scan.

## UMAP plot of the multiplexed scRNA-seq experiment described in Figure 1. Within the whole dataset, an unsupervised Leiden clustering identified 15 clusters. For cell-type attribution, we performed a per-cluster similarity search against > 15 million annotated single-cell transcriptomes from published studies using CellSearch (Bioturing). The dot plot shows the number of high-quality matches to published annotated cell populations for each cluster. The matching cell types were monocytes, microglia, macrophages, neutrophils, and DCs.

1. Expression intensity projections of canonical marker genes for DCs, macrophages, and neutrophils.
2. GM-CSF BM cells were cultured with heme (300 µM) or vehicle. On day 7, the cells were harvested, pulsed with ovalbumin (150 µg/ml), washed to remove any remaining heme, and then incubated with CFSE-labeled naïve CD8^+^ T cells isolated from OT-1 mice. The proliferation of CD8^+^ T cells was assessed by evaluating the degree of CFSE dilution measured by flow cytometry after three days in coculture. Left: Representative data for cells gated based on positive CD8 expression are shown. Right: Cumulative T cell proliferation data from four independent coculture experiments.
3. CFSE-labeled CD4^+^ T cells isolated from the spleen of OT-2 mice and OVA_323-339_ (10 μgr/ml)-pulsed GM-CSF BM cells that were exposed to heme or vehicle were injected intravenously via the tail vein into Rag1^-/-^ mice expressing the CD45.1 antigen. Three days later, the spleens were harvested and the level of CFSE dilution was measured by flow cytometry. Left: representative contour plots showing the expression of CD4 and CD45.1. Right: CFSE dilution in cells gated based on CD45.1^+^ CD4^+^ T cells (red boxed).
4. Cumulative T cell proliferation data from three independent experiments.

The data in F are presented as the means ± SDs. t-test (E, G); n.s. = not significant, **P* ≤ 0.05, ***P* ≤ 0.01, ****P* ≤ 0.001, *****P* ≤ 0.0001.

**Supplementary Figure 2**

1. GSEA of the differentially expressed genes in M-CSF or GM-CSF BM cultures (heme versus control) obtained by scRNA-seq analysis with annotation of all gene sets (rows) and the corresponding false discovery rate (referring to Figure 1). The heatmaps represent the magnitude of the running enrichment score per gene set category (red indicates positive enrichment, and blue indicates negative enrichment). The GSEA is based on the hallmark gene sets of the Molecular Signature Database (MSigDB).
2. Multiplexed scRNA-seq experiment of BM cells supplemented with GM-CSF as described in Figure 2. Top: the UMAP plots represent the expression intensity of genes associated with heme transport, heme metabolism, iron storage, and iron export. Bottom: UMAP plot showing the three terminal differentiated cell types as described in Figure 2B.

**Supplementary Figure 3**

1. GSEA of differentially expressed genes (heme exposure versus vehicle) in GM-CSF BM cultures at different timepoints of heme exposure (24, 72, 96 h). The heatmaps represent the running enrichment score per gene set category (red for positive enrichment, blue for negative enrichment). Each row represents one gene set category. The GSEA is based on the hallmark gene sets of the Molecular Signature Database (MSigDB).

**Supplementary Figure 4**

## NRF2 expression in whole cells and the nuclei fractions of human HEK293 cells exposed to heme (300 µM) for 0, 2, 4, and 6 hours measured by western blot analysis.

1. Outline of the multiplexed scRNA-seq experiment of GM-CSF BM cultures from Nrf2^+/+^ and Nrf2^-/-^ mice. The cells were treated on day 3 with vehicle, heme (300 µM) or ML-334 (10 µM). On day 7, each sample was tagged with DNA-barcoded antibodies and pooled for sequencing and downstream analysis.
2. Correlation plot of the ranked GSEA enrichment scores for heme-regulated and ML-334-regulated gene sets. GSEA was performed using differential gene expression data from scRNA-seq analysis of Nrf2^+/+^GM-CSF BM cultures treated with heme or ML-334 versus vehicle.
3. Flow cytometry contour plots of GM-CSF BM cultures from Hmox1^fl/fl^ Cre^neg^  (wild-type) , Hmox1^fl/fl^ Ubc-Cre/ERT2^pos^, and Hmox1^fl/fl^ Vav-Cre^pos^ mice . The cells were treated on day 3 with vehicle or heme (50 µM). The induction of target gene recombination in Hmox1^fl/fl^ Ubc-Cre/ERT2^pos^ was induced by 4-hydrotamoxifen treatment.
4. Hierarchical clustering analysis of normalized mRNA expression intensities for Nrf2 target genes in Hmox1^fl/fl^ Cre^neg^ , Hmox1^fl/fl^ Ubc-Cre/ERT2^pos^ , and Hmox1^fl/fl^ Vav-Cre^pos^ GM-CSF BM cells treated from day 3 to day 7 with 50 µM heme (red) or vehicle (blue) and analyzed by qRT-PCR.
5. Flow cytometry contour plots of GM-CSF BM cultures from Keap1^fl/fl^ Cre^neg^ and Keap1^fl/fl^ Ubc-Cre/ERT2^pos^ . The induction of target gene recombination was induced by 4-hydrotamoxifen treatment.

**Supplementary Figure 5**

1. UMAP plots of pooled scRNA-seq data from two sequencing experiments with spleen cell suspensions from an SCD mouse and a wild-type mouse. Before sequencing, the spleen cells were enriched for macrophages using anti-F4/80 antibody coated magnetic Dynabeads. For the whole dataset, unsupervised Leiden clustering identified 14 clusters that were attributed to six cell types (see Supplementary Figure 6).
2. Left: UMAP plots of the data split by strain (wild-type versus SCD). Right: The stack bar charts illustrate each cell type as a proportion of the whole number of cells per strain suggesting a massive expansion of red pulp macrophages (RPMs) and erythropoietic island macrophages (EBIs).
3. Top: Expression intensity projections of canonical marker genes for red pulp macrophages and DCs. Bottom: Expression intensity projections of genes associated with heme metabolism and iron metabolism.

**Supplementary Figure 6**

Cell type attribution of the scRNA-seq experiment displayed in Supplementary Figure 5

1. UMAP plots of pooled scRNA-seq data from two sequencing experiments with spleen cell suspensions from an SCD mouse and a wild-type mouse. Before sequencing, spleen cells were enriched for macrophages using anti-F4/80 antibody coated magnetic Dynabeads. For the whole dataset, unsupervised Leiden clustering identified 14 clusters that were attributed to red pulp macrophages (RPM), erythroblastic island macrophages (EBIs), DCs, B lymphocytes, monocytes, and neutrophils.
2. Cell-type attribution. The dot plots show the normalized mean expression intensity (color) and the fraction of positive cells (size) for selected canonical marker genes in each Leiden cluster (top) and each identified cell type (bottom). The dendrograms show the hierarchical clustering based on the PCA components between the clusters (top) and the different cell types (bottom).

**Supplementary Figure 7**

1. scRNA-seq data of spleen cell suspensions from SCD and wild-type mice negatively enriched for DCs (see Figure 5). UMAP plots of the pooled scRNA-seq data (SCD and wild-type) colored by macrostates. The arrows indicate the calculated velocity.
2. Dot plot showing the mean expression levels of selected signature genes in each macrostate. The dot size indicates the fraction of cells expressing the genes, and the color indicates the per-gene normalized mean expression. The dendrogram represents the hierarchical clustering results based on the PCA components between the macrostates.

## UMAP plots of the pooled scRNA-seq data (SCD and wild-type) colored by cell types.

## The UMAP projections display the overall differentiation potential of every cell (left) and the transition probability of each cell into one of the six terminal states (right). For each terminal state a UMAP projection color coded representing the probability of each cell reaching this terminal state is shown.

1. Cumulative absorption probabilities across the whole experiment per cell type and genotype point to a reduced absorption probability for DCs in the SCD mouse. This plot aggregates and summarizes absorption probabilities from a single cell level for each experimental condition.

**Supplementary Figure 8**

## Flow cytometry contour plots of splenic cell suspensions from SCD, Spta^sph/sph^ mice, and their littermates. Representative cells were gated based on positive CD45 expression (cumulative data shown in Figure 6F). Percentage of DCs identified as CD11c and MHC class II positive cells are highlighted in red.

1. Plasma concentrations of IL12p70 and CXCL10 in Spta^sph/sph^ mice and wild-type littermates treated with saline (control) or an agonistic anti-CD40 antibody measured with a Bio-Plex assay.
2. Cumulative in vitro T cell proliferation data from three independent experiments (corresponding to Figure 6G).

The data in B and C are presented as the means ± SDs. Each dot represents one independent experiment. one-way ANOVA with Tukey’s multiple comparison test (B). t-test (C). n.s. = not significant, **P* ≤ 0.05, ***P* ≤ 0.01, ****P* ≤ 0.001, *****P* ≤ 0.0001.
