# Supplementary material for: Heme-stress activated NRF2 skews fate trajectories of bone marrow cells from dendritic cells towards red pulp-like macrophages in hemolytic anemia
Source: Cell Death Differ. 2022 Jan 14;29(8):1450–65. doi: 10.1038/s41418-022-00932-1 (PMC9345992; doi:10.1038/s41418-022-00932-1)
Supplement: Supplementary file 2 — Supplementary resource table [file 41418_2022_932_MOESM2_ESM.docx]

| **Resource Table** | | |
| --- | --- | --- |
| **Antibodies** | | |
| **Reagent or Resource** | **Source** | **Identifier** |
| CD115 BV605 | BioLegend | Cat # 135517 |
| CD115 PE-Cy7 | BioLegend | Cat # 135523 |
| CD11b APC-Cy7 | BioLegend | Cat # 101226 |
| CD11c APC | BioLegend | Cat # 117310 |
| CD11c PE | BD Pharmingen | Cat # 553802 |
| CD19 FITC | BD Pharmingen | Cat # 553785 |
| CD25 PE | BD Pharmingen | Cat # 558642 |
| CD3 BUV395 | BD Pharmingen | Cat # 740268 |
| CD4 PE | BioLegend | Cat # 100408 |
| CD4 eFluor 450 | ThermoFisher | Cat # 48-0041-82 |
| CD45 PE-Cy7 | BD Pharmingen | Cat # 552848 |
| CD45 Brilliant Violet 421 | BioLegend | Cat # 103134 |
| CD45.1 BV605 | BioLegend | Cat # 110739 |
| CD45.2 Pacific Blue | BioLegend | Cat # 109820 |
| CD69 APC | BioLegend | Cat # 104514 |
| F480 BV605 | BioLegend | Cat # 123133 |
| Ly6G PE | BioLegend | Cat # 127608 |
| Ly6G APC | BioLegend | Cat # 127614 |
| MHC2 BV421 | BD Pharmingen | Cat # 562564 |
| MHC2 Alexa Fluor 647 | BD Pharmingen | Cat # 562367 |
| CD8 PE | BioLegend | Cat # 100708 |
| TotalSeq™ B0302 Anti-mouse Hashtag antibodies | Biolegend | Cat # 155833 |
| TotalSeq™ B0303 Anti-mouse Hashtag antibodies | Biolegend | Cat # 155835 |
| TotalSeq™ B0304 Anti-mouse Hashtag antibodies | Biolegend | Cat # 155837 |
| TotalSeq™ B0305 Anti-mouse Hashtag antibodies | Biolegend | Cat # 155839 |
| TotalSeq™ B0306 Anti-mouse Hashtag antibodies | Biolegend | Cat # 155841 |
| TotalSeq™ B0307 Anti-mouse Hashtag antibodies | Biolegend | Cat # 155843 |
| TotalSeq™ B0308 Anti-mouse Hashtag antibodies | Biolegend | Cat # 155845 |
| Agonistic anti-CD40 antibody | InVivoPLus | Cat # BP0016-2 |
| Nrf2 polyclonal antibody | Thermofisher | Cat # PA5-27882 |
| Goat Anti-Rabbit IgG (H + L)-HRP Conjugate | Biorad | Cat # 1706515 |
| Bio-Plex Pro™ Mouse Cytokine IP-10/ CXCL10 | Bio-rad | Cat # 12002244 |
| Mouse CD25/IL-2 R alpha DuoSet ELISA | R&D Systems | Cat # DY2438 |
| Bio-Plex Pro™ Mouse Cytokine IL12p70 | Bio-rad | Cat # 171G5011M |
| **Dyes** | | |
| **Reagent or Resource** | **Source** | **Identifier** |
| LIVE/DEAD™ Fixable Near-IR Dead Cell Stain Kit | Invitrogen | Cat # L10119 |
| Thiazole-orange | Sigma Aldrich | Cat # 390062 |
| **Chemicals, Peptides, and Recombinant proteins** | | |
| **Reagent or Resource** | **Source** | **Identifier** |
| EndoFit Ovalbumin (Chicken egg albumin; for in vivo use) | InvivoGen | Cat # 17E10-MM |
| Ovalbumin (323-339) (chicken, Japanese quail) | Sigma Aldrich | Cat # O1641 |
| RA-839 | Tocris | Cat # 5707 |
| ML-334 | Tocris | Cat # 5625 |
| Recombinant Murine GM-CSF | Peprotech | Cat # 315-03 |
| Recombinant Murine M-CSF | Peprotech | Cat # 315-02 |
| 20% Human Serum Albumin | CSL Berhing AG | Cat # 3665734 |
| Hemin | Frontier Scientific | Cat # H651-9 |
| SnMP (tin mesoporphyrin) | Frontiers Scientific | Cat # SnM321 |
| Phosphate buffered Saline (PBS) | Gibco | Cat # 10010-015 |
| Penicillin-Streptomycin | Thermo Fisher | Cat # 15140-122 |
| RPMI Medium | Gibco | Cat # 11835-063 |
| Glutamax | Gibco | Cat # 35050-061 |
| MACS Buffer BSA Stock Solution | Miltenyi Biotec | Cat #130-091-376 |
| Dulbecco's MEM | Merck | Cat # 1469C |
| RBC Lysis Buffer (10X) | Biolegend | Cat #420301 |
| Fetales bovines Serum | Gibco | Cat # 10270-106 |
| Collagenase Type IV | Stemcell | Cat # 7427 |
| **Critical Commercial Assays** | | |
| **Reagent or Resource** | **Source** | **Identifier** |
| CellTrace™ Far Red Cell Proliferation Kit, for flow cytometry | ThermoFisher | Cat # C34564 |
| CellTrace™ Violet Cell Proliferation Kit, for flow cytometry | ThermoFisher | Cat # C34557 |
| UltraComp eBeads™ Compensation Beads | ThermoFisher | Cat # 01-2222-42 |
| Tru Stain FcxTmPLUS CD16/32, clone S17011E Isotype Rat IgG2b | Biolegend | Cat # 156604 |
| Dynabeads™ Mouse DC (Dendritic Cell) Enrichment | Invitrogen | Cat # 11429D |
| Dynabeads™ FlowComp™ Mouse CD4 Kit | Invitrogen | Cat # 11461D |
| MagniSort™ Mouse F4/80 Positive Selection Kit | Invitrogen | Cat # 8802-6863-74 |
| Lineage Cell Depletion Kit, mouse for 1×109 total cells | Miltenyi Biotec | Cat # 130-090-858 |
| Dynabeads™ FlowComp™ Mouse CD8 Kit | ThermoFisher | Cat # 11462D |
| **Experimental Models: Organisms/Strains** | | |
| **Reagent or Resource** | **Source** | **Identifier** |
| Mouse: C57BL/6NCrl | Charles River | 027C57BL/6 |
| Mouse: Hba<tm1Paz> Hbb<tm1Tow> Tg(HBA-HBBs)41Paz/J | The Jackson Laboratory | Jax # 003342 |
| Mouse: Spta1 B6.C3-Spta1<sph>/BrkJ | The Jackson Laboratory | Jax # 000450 |
| Mouse: WB.C3-Spta1 sph /BrkJ | The Jackson Laboratory | Jax # 000454 |

Mouse: B6.129P2-Keap1<tm2Mym> Riken RBRC09595
