# Supplementary figures and images for: Heme-stress activated NRF2 skews fate trajectories of bone marrow cells from dendritic cells towards red pulp-like macrophages in hemolytic anemia
Source: Cell Death Differ. 2022 Jan 14;29(8):1450–65. doi: 10.1038/s41418-022-00932-1 (PMC9345992; doi:10.1038/s41418-022-00932-1)

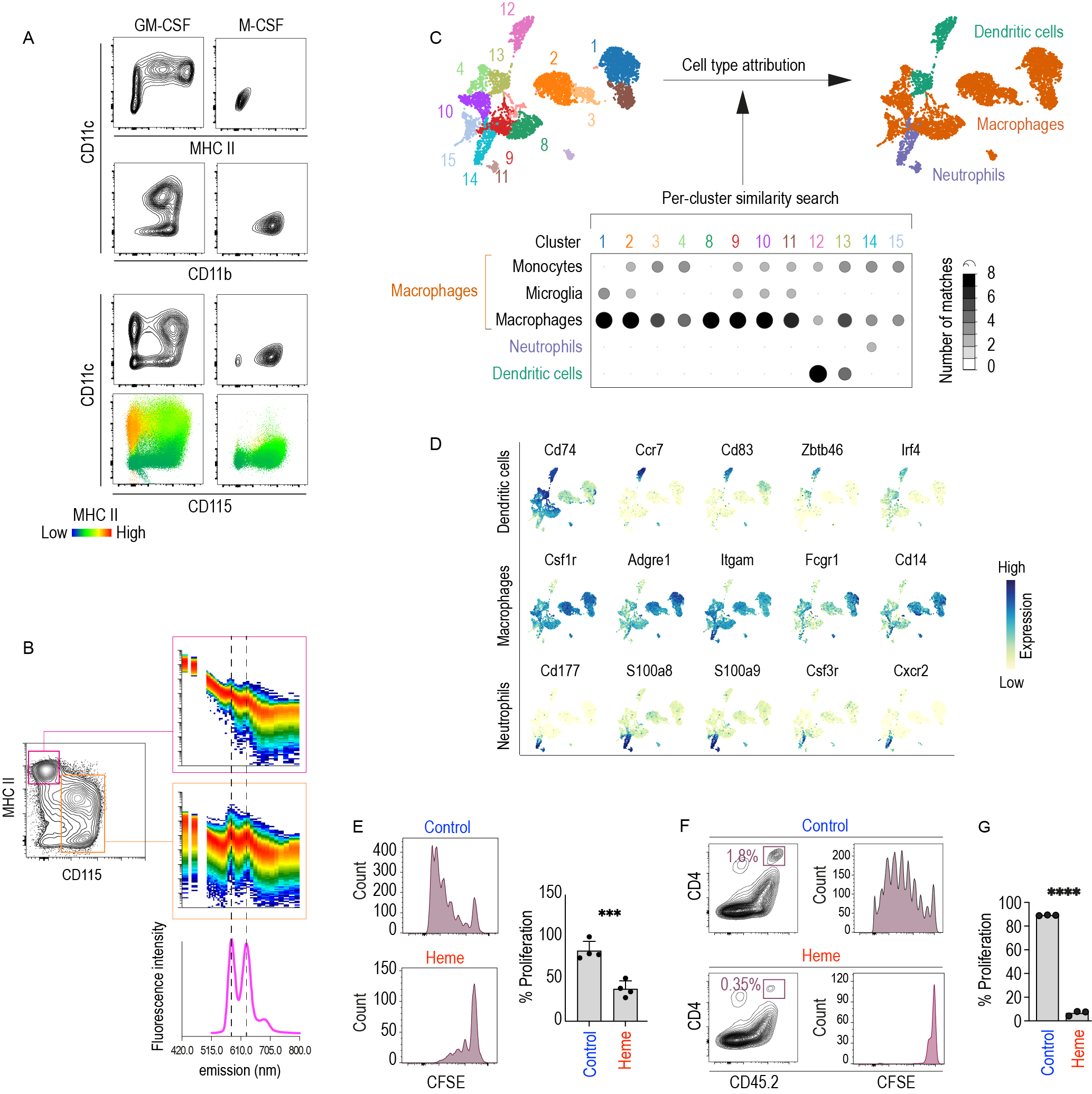

Supplement: Supplementary file 3 — Supplementary Figure 1 [file 41418_2022_932_MOESM3_ESM.png]

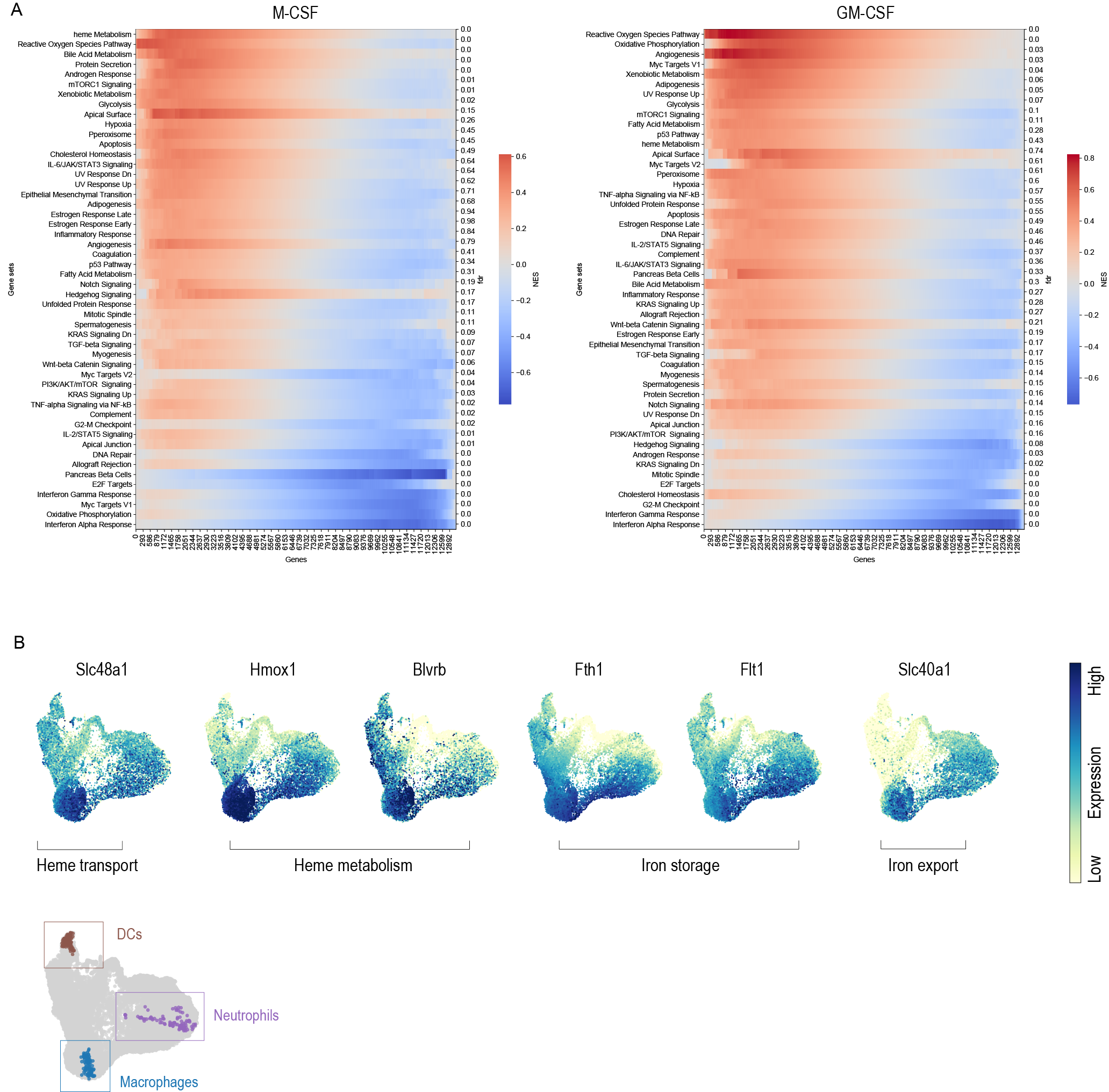

Supplement: Supplementary file 4 — Supplementary Figure 2 [file 41418_2022_932_MOESM4_ESM.png]

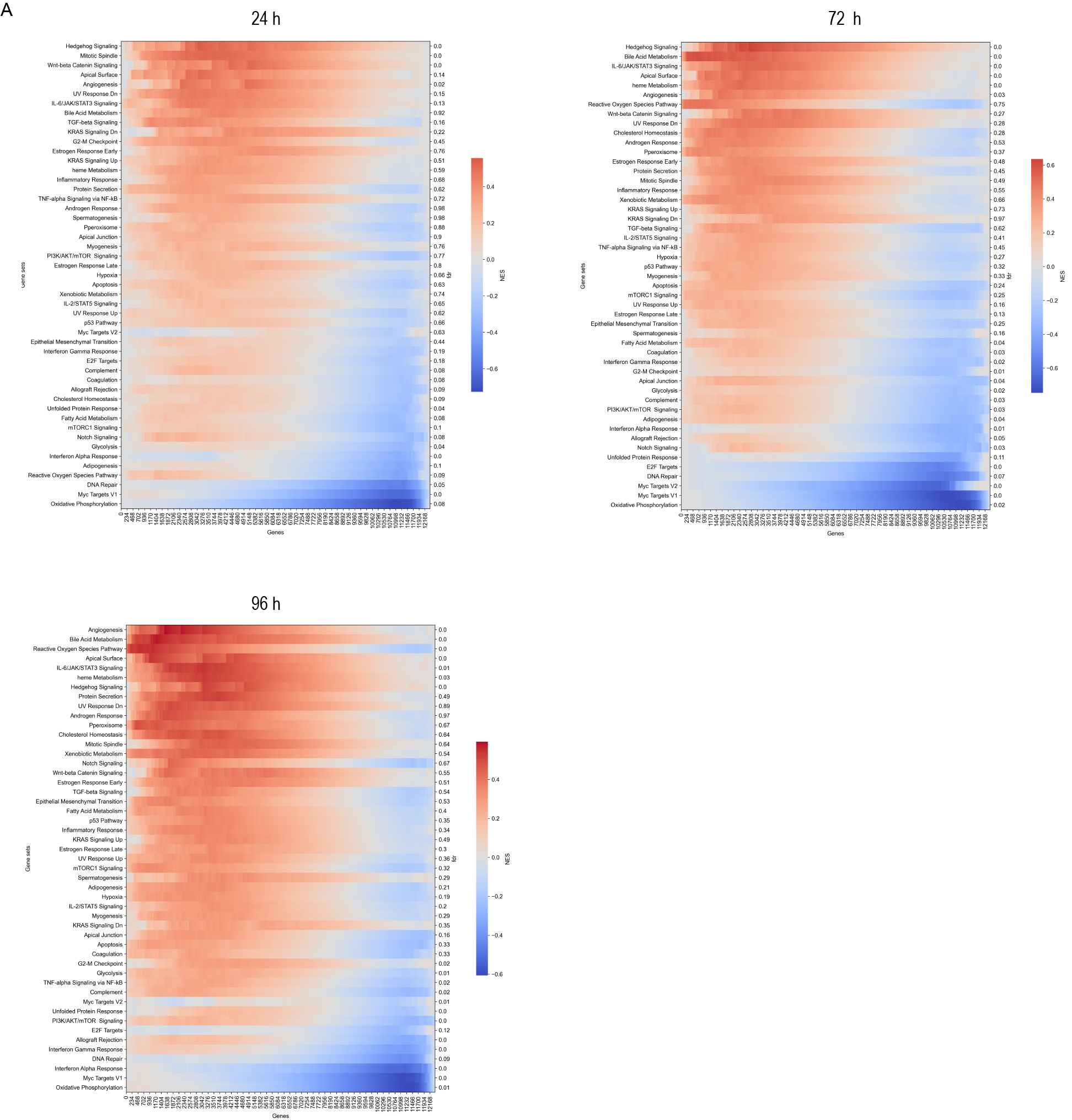

Supplement: Supplementary file 5 — Supplementary Figure 3 [file 41418_2022_932_MOESM5_ESM.png]

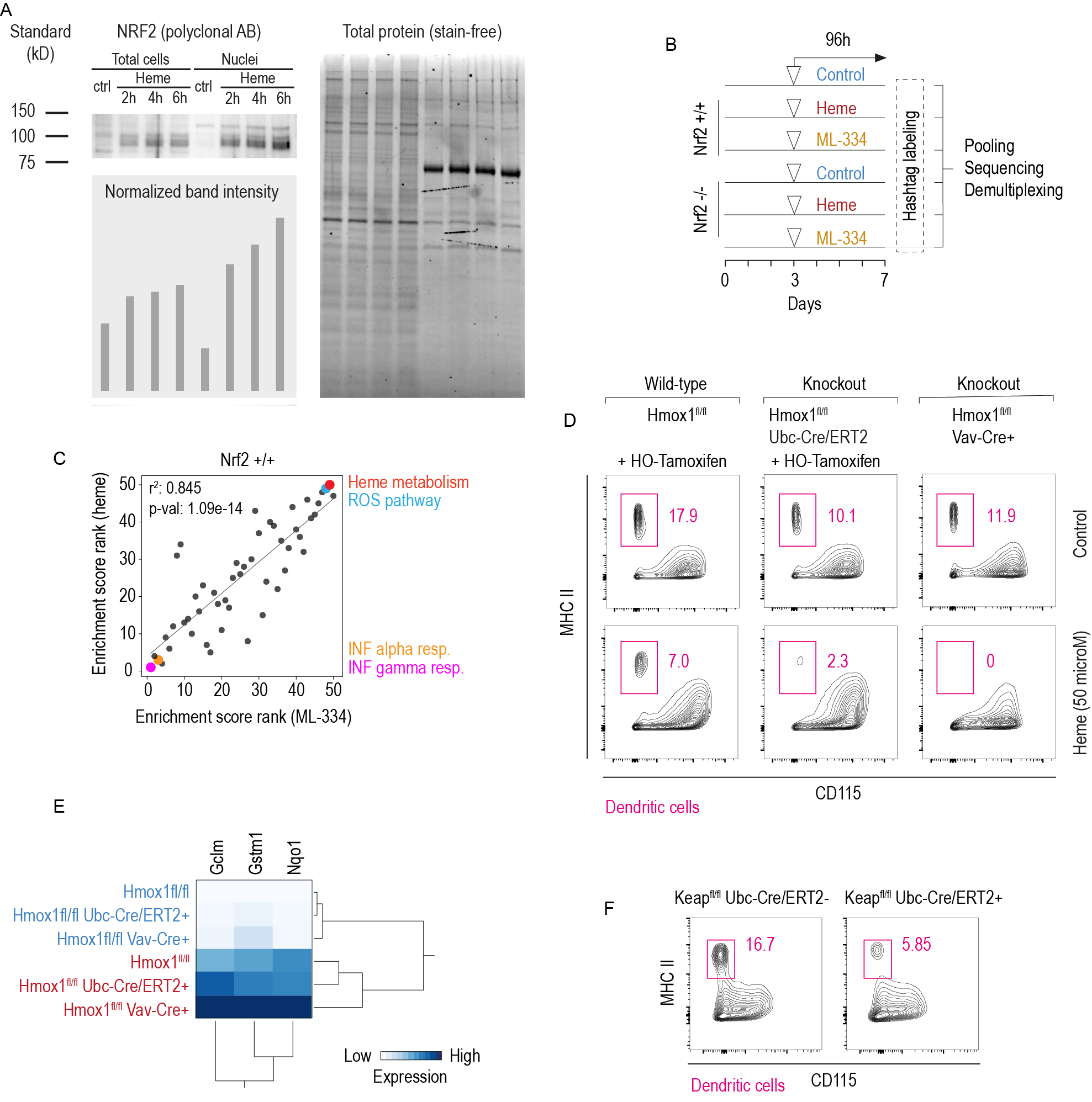

Supplement: Supplementary file 6 — Supplementary Figure 4 [file 41418_2022_932_MOESM6_ESM.png]

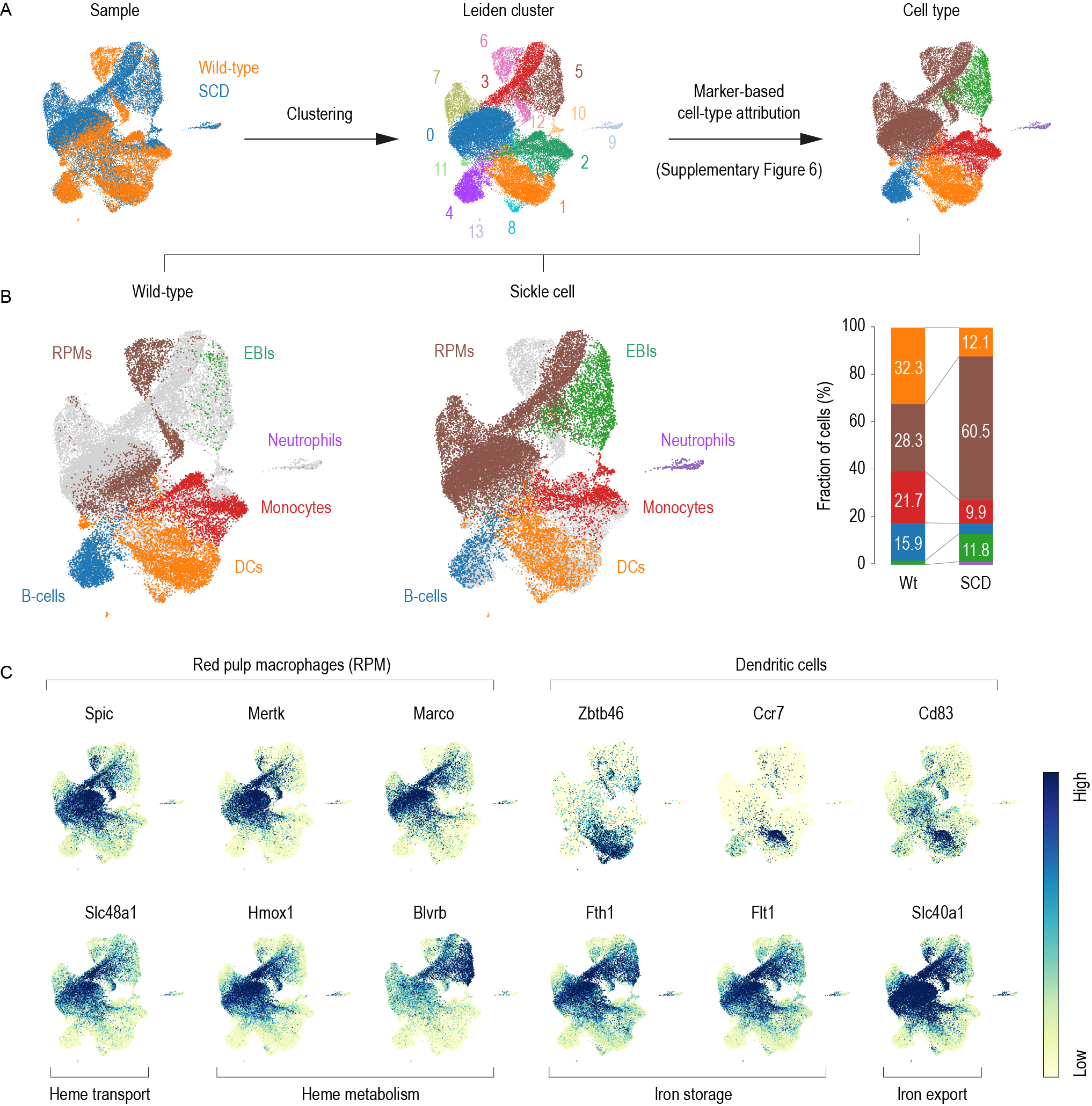

Supplement: Supplementary file 7 — Supplementary Figure 5 [file 41418_2022_932_MOESM7_ESM.png]

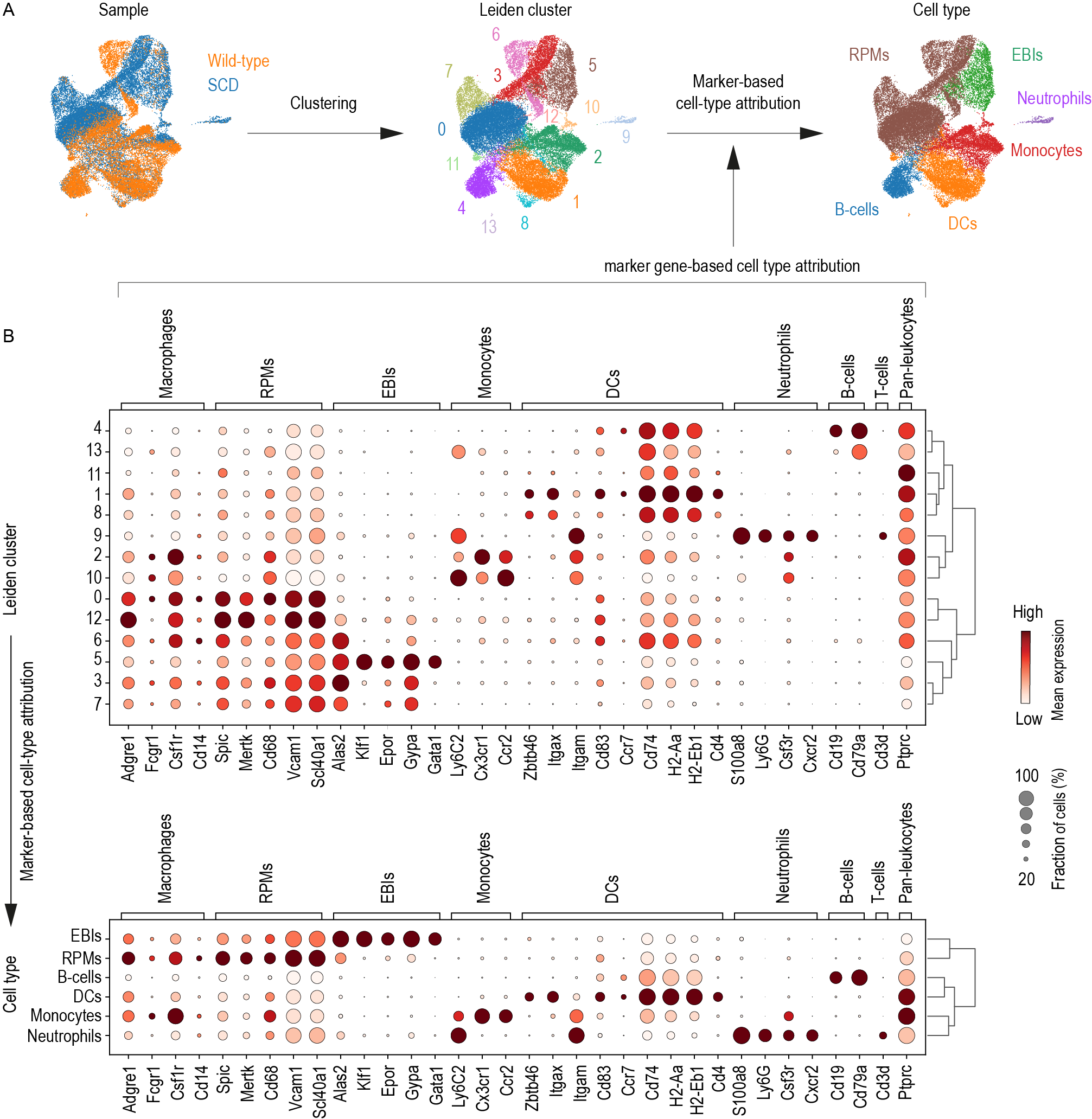

Supplement: Supplementary file 8 — Supplementary Figure 6 [file 41418_2022_932_MOESM8_ESM.png]

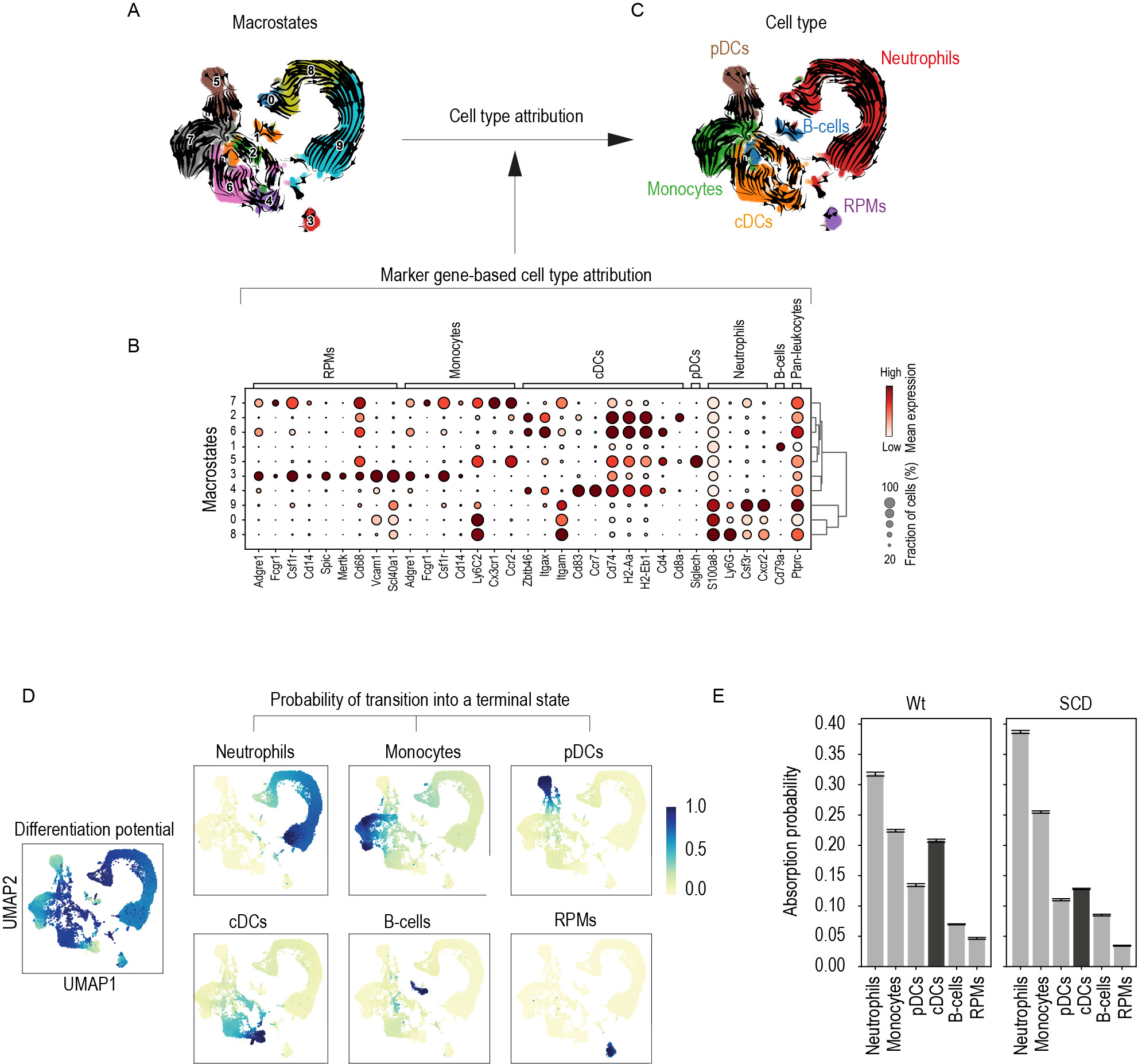

Supplement: Supplementary file 9 — Supplementary Figure 7 [file 41418_2022_932_MOESM9_ESM.png]

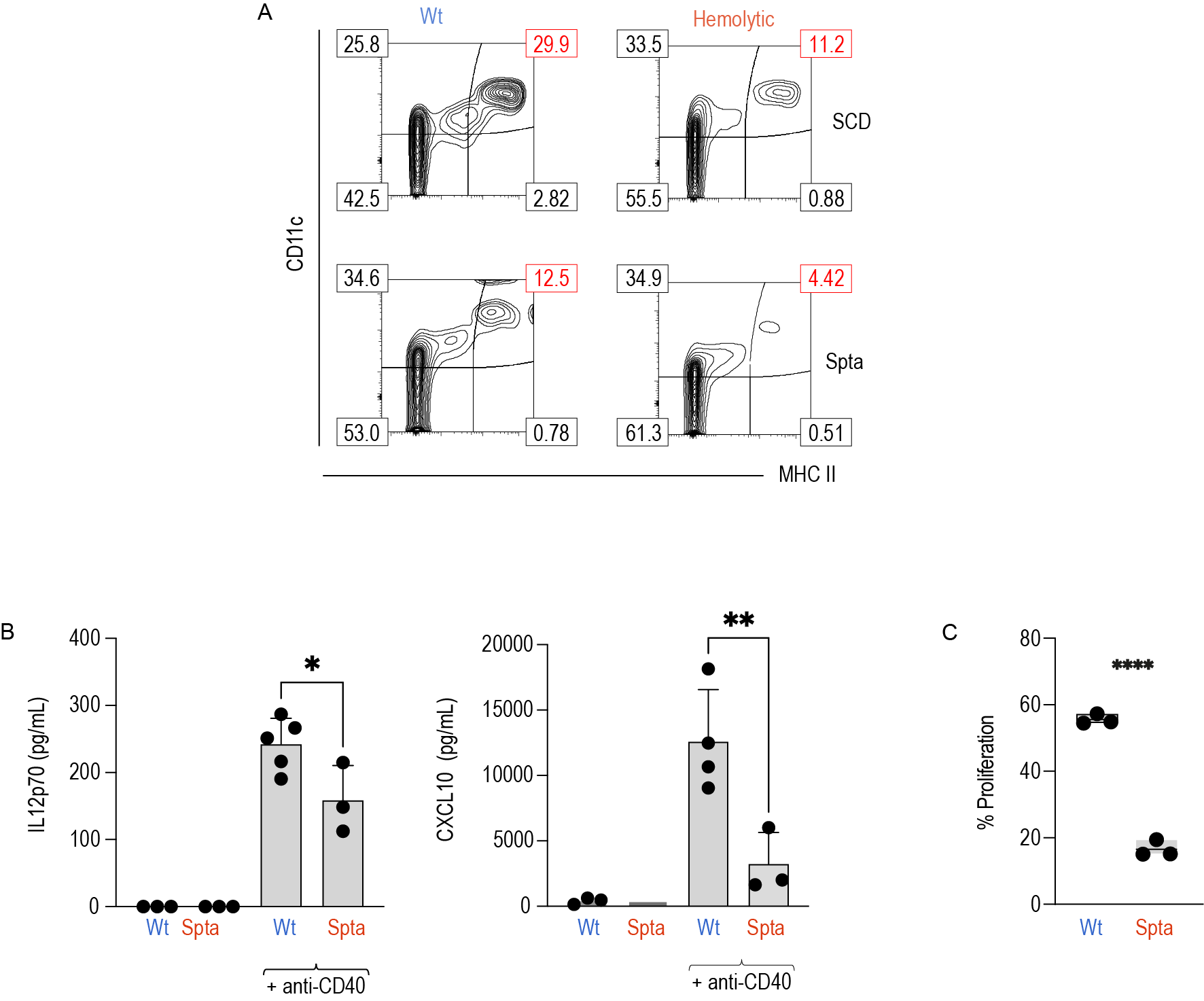

Supplement: Supplementary file 10 — Supplementary Figure 8 [file 41418_2022_932_MOESM10_ESM.png]
