# Supplementary material for: Heme-stress activated NRF2 skews fate trajectories of bone marrow cells from dendritic cells towards red pulp-like macrophages in hemolytic anemia
Source: Cell Death Differ. 2022 Jan 14;29(8):1450–65. doi: 10.1038/s41418-022-00932-1 (PMC9345992; doi:10.1038/s41418-022-00932-1)
Supplement: Supplementary file 12 — authorship form [file 41418_2022_932_MOESM12_ESM.pdf]

SPRINGER NATURE

Change of authorship request form - Journals (pre-acceptance)

Important information. Please read.

- This form should be used by authors to request any change in authorship (adding/deleting authors) including changes in corresponding authors. This form should not be used for name changes. Please fully complete all sections. Use black ink and block capitals and provide each author's full name with the given name first followed by the family name.

- By signing this declaration, all authors guarantee that the order of the authors are in accordance with their scientific contribution, if applicable as different conventions apply per discipline, and that only authors have been added who made a meaningful contribution to the work.

- Please note, in author collaborations where there is formal agreement for representing the collaboration, it is sufficient for the representative or legal guarantor (usually the corresponding author) to complete and sign the Authorship Change Form on behalf of all authors, **next to the added/removed author(s)**. **(Complete Section 3, followed by Section 6.)**
  In author collaborations where there is no formal agreement for representing the collaboration and **there are more than 10 authors**, one may sign for all, provided the signer appends correspondence that attests that each of the authors have agreed to the change **and the added/removed authors sign the form**. **(Complete Section 3, followed by Section 6.)**

- Please note, we cannot investigate or mediate any authorship disputes. If you are unable to obtain agreement from all authors (including those who you wish to be removed) you must refer the matter to your institution(s) for investigation. Please inform us if you need to do this.

- If you are not able to return a fully completed form within **30 days** of the date that it was sent to the author requesting the change, we may have to withdraw your manuscript. We cannot publish manuscripts where authorship has not been agreed by all authors (including those who have been removed).

- Incomplete forms will be rejected.

- Please return/upload this form, fully completed, to the Journals Editorial Office. The Journal and/or Publisher will consider the information you have provided to decide whether to approve the proposed change in authorship. We may decide to contact your institution for more information or undertake a further investigation, if appropriate, before making a final decision.

SPRINGER NATURE Springer Nature is one of the world's leading global research, educational and professional publishers, created in May 2015 through the combination of Nature Publishing Group, Palgrave Macmillan, Macmillan Education and Springer Science+Business Media.

SPRINGER NATURE

Change of authorship request form - Journals (pre-acceptance)

Section 1: Please provide the current title of manuscript

Manuscript ID no.: CDD-21-1587RR

Title: Heme-stress activated NRF2 skews....

Section 2: Please provide the previous authorship, in the order shown on the manuscript before the changes were introduced. Please indicate the corresponding author by adding (CA) behind the name.

	First name(s)	Family name	ORCID or SCOPUS id, if available
1st author	Florence	Vallelian (CA)	
2nd author	Raphael	Buzzi	
3rd author	Marc	Pfefferle	
4th author	Ayla	Yalamanoglu	
5th author	Andreas	Wassmer	
6th author	Thomas	Gentinetta	
7th author	Kerstin ~~Hans~~	Hansen	
8th author	Rok	Humar	
9th author	Nadja	Schulthess	
10th author	Dominik	Schaer	

Please use an additional sheet if there are more than 10 authors.

SPRINGER NATURE Springer Nature is one of the world's leading global research, educational and professional publishers, created in May 2015 through the combination of Nature Publishing Group, Palgrave Macmillan, Macmillan Education and Springer Science+Business Media.

SPRINGER NATURE

Change of authorship request form - Journals (pre-acceptance)

Section 3: Please provide a justification for change. Please use this section to explain your reasons for changing the authorship of your manuscript, e.g. what necessitated the change in authorship? Please refer to the (journal) policy pages for more information about authorship. Please explain why omitted authors were not originally included and/or why authors were removed on the submitted manuscript.

- additional experiments performed by the new authors for the revision of the paper

Section 4: Proposed new authorship. Please provide your new authorship list in the order you would like it to appear on the manuscript. Please indicate the corresponding author by adding (CA) behind the name. If the Corresponding Author has changed, please indicate the reason under section 3.

	First name(s)	Family name (this name will appear in full on the final publication and will be searchable in various abstract and indexing databases)	Affiliated institute	E-mail address
1st author	Florence	Vallelian (CA)	University of Zurich	
2nd author	Raphael	Buzzi	University of Zurich	
3rd author	Marc	Pfefferle	University of Zurich	
4th author	Ayla	Yalamanoglu	University of Zurich	
5th author	Irina	Dubach	University of Zurich	
6th author	Andreas	Wassmer	CSL Behring	
7th author	Thomas	Gentinetta	CSL Behring	
8th author	Kerstin	Hansen	University of Zurich	
9th author	Rok	Humar	University of Zurich	
10th author	Nadja	Schulthess	University of Zurich	

Please use an additional sheet if there are more than 10 authors.

11 Christian Schaer University of Zurich

12 Dominik Schaer University of Zurich

SPRINGER NATURE Springer Nature is one of the world's leading global research, educational and professional publishers, created in May 2015 through the combination of Nature Publishing Group, Palgrave Macmillan, Macmillan Education and Springer Science+Business Media.

SPRINGER NATURE **Change of authorship request form - Journals** (pre-acceptance)

Section 5: Author contribution, Acknowledgement and Disclosures. Please use this section to provide a new disclosure statement and, if appropriate, acknowledge any contributors who have been removed as authors and ensure you state what contribution any new authors made (if applicable per the journal or book (series) policy). Please ensure these are updated in your manuscript - after approval of the change(s) - as our production department will not transfer the information in this form to your manuscript.

New acknowledgements:

no new acknowledgments

New Disclosures (financial and non-financial interests, funding):

no new disclosures

New Author Contributions statement (if applicable per the journal policy):

Irina Dubach: performed experiments

Christian Schaer: performed experiments and analyzed data

State 'Not applicable' if there are no new authors.

SPRINGER NATURE Springer Nature is one of the world's leading global research, educational and professional publishers, created in May 2015 through the combination of Nature Publishing Group, Palgrave Macmillan, Macmillan Education and Springer Science+Business Media.

SPRINGER NATURE

Change of authorship request form - Journals (pre-acceptance)

Section 6: Declaration of agreement. *All* authors, unchanged, new and removed *must* sign this declaration.
(NB: Please print the form, (docu)-sign and return/upload a scanned copy. Please note that signatures that have been inserted as an image file are acceptable as long as it is handwritten. Typed names in the signature box are unacceptable.) *** Please delete as appropriate. Delete all of the bold if you were on the original authorship list and are remaining as an author.**

	First name	Family name		Signature	Date
1st author	Florence	Vallelian	I agree to the proposed new authorship shown in section 4 **/and the addition/removal*of my name to the authorship list** /and the proposed change in corresponding author	[illegible]	16.12.2021
2nd author	Raphael	Buzzi	I agree to the proposed new authorship shown in section 4 **/and the addition/removal*of my name to the authorship list** /and the proposed change in corresponding author	[illegible]	16.12.21
3rd author	Marc	Pfefferle	I agree to the proposed new authorship shown in section 4 **/and the addition/removal*of my name to the authorship list** /and the proposed change in corresponding author	[illegible]	16.12.21
4th authors	Ayla	Yalamanoglu	I agree to the proposed new authorship shown in section 4 **/and the addition/removal*of my name to the authorship list** /and the proposed change in corresponding author	[illegible]	17.12.21
5th author	Irina	Dubach	I agree to the proposed new authorship shown in section 4 **/and the addition/removal*of my name to the authorship list** /and the proposed change in corresponding author	[illegible]	16.12.21
6th author	Andreas	Wassmer	I agree to the proposed new authorship shown in section 4 **/and the addition/removal*of my name to the authorship list** /and the proposed change in corresponding author	[illegible]	17.12.21
7th author	Thomas	Gentinetta	I agree to the proposed new authorship shown in section 4 **/and the addition/removal*of my name to the authorship list** /and the proposed change in corresponding author	[illegible]	17.12.21

SPRINGER NATURE Springer Nature is one of the world's leading global research, educational and professional publishers, created in May 2015 through the combination of Nature Publishing Group, Palgrave Macmillan, Macmillan Education and Springer Science+Business Media.

SPRINGER NATURE

Change of authorship request form - Journals (pre-acceptance)

Section 6: Declaration of agreement. *All* authors, unchanged, new and removed *must* sign this declaration.
(NB: Please print the form, (docu)-sign and return/upload a scanned copy. Please note that signatures that have been inserted as an image file are acceptable as long as it is handwritten. Typed names in the signature box are unacceptable.) *** Please delete as appropriate. Delete all of the bold if you were on the original authorship list and are remaining as an author.**

	First name	Family name		Signature	Date
~~1st author~~ 11	Christian	Schaer	I agree to the proposed new authorship shown in section 4 **/and the addition/removal*of my name to the authorship list** /and the proposed change in corresponding author		12/17/2021
~~2nd author~~ 12	Dominik	Schaer	I agree to the proposed new authorship shown in section 4 **/and the addition/removal*of my name to the authorship list** /and the proposed change in corresponding author		12/16/2021
3rd author			I agree to the proposed new authorship shown in section 4 **/and the addition/removal*of my name to the authorship list** /and the proposed change in corresponding author		
4th authors			I agree to the proposed new authorship shown in section 4 **/and the addition/removal*of my name to the authorship list** /and the proposed change in corresponding author		
5th author			I agree to the proposed new authorship shown in section 4 **/and the addition/removal*of my name to the authorship list** /and the proposed change in corresponding author		
6th author			I agree to the proposed new authorship shown in section 4 **/and the addition/removal*of my name to the authorship list** /and the proposed change in corresponding author		
7th author			I agree to the proposed new authorship shown in section 4 **/and the addition/removal*of my name to the authorship list** /and the proposed change in corresponding author		

SPRINGER NATURE Springer Nature is one of the world's leading global research, educational and professional publishers, created in May 2015 through the combination of Nature Publishing Group, Palgrave Macmillan, Macmillan Education and Springer Science+Business Media.

SPRINGER NATURE

Change of authorship request form - Journals (pre-acceptance)

	First name	Family name		Signature	Date
8th author	Kerstin	Hansen	I agree to the proposed new authorship shown in section 4 **/and the addition/removal*of my name to the authorship list** /and the proposed change in corresponding author	[illegible]	16.12.2021
9th author	Rok	Humar	I agree to the proposed new authorship shown in section 4 **/and the addition/removal*of my name to the authorship list** /and the proposed change in corresponding author	[illegible]	
10th author	Nadja	Schultheiss	I agree to the proposed new authorship shown in section 4 **/and the addition/removal*of my name to the authorship list** /and the proposed change in corresponding author	[illegible]	16.12.2021

Please use an additional sheet if there are more than 10 authors.

In case of author collaborations with formal agreement:

	Name of consortium/consortia	First name	Family name		Signature	Date
Representative/legal guarantor				I agree to the proposed new authorship shown in section 4 **/and the addition/removal*of my name to the authorship list** /and the proposed change in corresponding author		

Both added/removed authors should complete the information in the first table under Section 6.

---- End of form ----

SPRINGER NATURE Springer Nature is one of the world's leading global research, educational and professional publishers, created in May 2015 through the combination of Nature Publishing Group, Palgrave Macmillan, Macmillan Education and Springer Science+Business Media.
